# The Role of EU Trade Secrets Law in the Data Economy: An Empirical Analysis

**DOI:** 10.1007/s40319-023-01325-8

**Published:** 2023-05-10

**Authors:** Tanya Aplin, Alfred Radauer, Martin A. Bader, Nicola Searle

**Affiliations:** 1 Dr.; Professor of Intellectual Property Law, Dickson Poon School of Law, King’s College London, London, UK; 2 Dr.; Head of Institute Business Administration and Management, IMC Krems, University of Applied Sciences, Krems an der Donau, Austria; 3 Dr.; Professor of Technology Management and Entrepreneurship, European and Swiss Patent Attorney, THI Business School, Technische Hochschule Ingolstadt, University of Applied Sciences, Ingolstadt, Germany; 4 Dr.; EPRSC Digital Economy Fellow and Senior Lecturer at Institute for Creative and Cultural Entrepreneurship, Goldsmiths, University of London, London, UK

**Keywords:** Trade secrets, Data economy, Data sharing, Confidential and commercially valuable data, Contract, Copyright, Database right, EU legal policy

## Abstract

This article draws on a recently completed study for the European Commission on trade secrets in the data economy. It distils the main findings of that Study and advances it by reflecting on and analyzing these findings in the context of existing legal, management and economics literature, as well as their implications for EU legal policymaking when it comes to trade secrets law. In order to facilitate data sharing, the article argues for a cautious approach, with very modest legislative reforms to the EU Trade Secrets Directive, instead preferring soft law and practical steps to be taken. There is, however, greater scope to reform legal regimes that are complementary to EU trade secrets law, such as the *sui generis* database right.

## Introduction

Data drives economies. In our now-digital economy, information is quickly digitized and circulated, and ubiquitous devices collect and generate data. The proliferation of data is astounding, with the prediction that the global volume of data will grow to 175 zettabytes by 2025.^1^ It has quickly become a key asset for firms and informs every aspect of a firm’s decisions making, from innovation to market strategies. Advances in technologies such as machine learning^2^ are poised to expand and entrench the power of data and its economic potential.

European policymakers have for several years sought to regulate how data is protected, shared and re-used through a raft of legislation. For example, the Database Directive,^3^ Digital Single Market Directive^4^ (with its explicit provisions on text and data mining), General Data Protection Regulation,^5^ Digital Markets Act,^6^ Digital Services Act^7^ and Data Governance Act,^8^ and proposed Data Act^9^ are all examples of “horizontal” means to address data sharing and protection issues across industries. There are also “vertical”, industry specific regulations to be considered, such as the Open Data Directive^10^ (that makes public sector and publicly-funded data re-usable); the Revised Payment Services Directive;^11^ data exclusivity in the pharmaceutical sector, whereby clinical trial test data of originator pharma firms is protected for an initial period of up to eight years;^12^ in the automotive sector, the Vehicle Repair and Maintenance Information^13^ and Vehicle Emissions Regulations;^14^ and, in the energy sector, the Energy Framework (Clean Energy for All Europeans Package).^15^

The Trade Secrets Directive^16^ (TSD), adopted in 2016, can be added to this complex assortment of regulation that frames the data economy. The Directive was introduced to address the problems of legal divergences in the protection of trade secrets in EU Member States and the benefit of such harmonisation was touted to be greater knowledge-exchange between businesses and increased incentives to engage in innovation-related activities in the EU, particularly on a cross-border basis.^17^ Although the TSD was not introduced with the concerns of data specifically in mind, the fact that it is a “generally applicable, technology neutral regime of protection”^18^ that protects “a wide range of know-how and business information”^19^ means that it is an important legal tool to consider in relation to the data economy.^20^

This article draws on a recently completed study for the European Commission on trade secrets in the data economy (“Study”).^21^ It distils the main findings of that in-depth Study and takes it further by reflecting on these findings and their implications for legal policymaking in relation to specific aspects of EU trade secrets law. It therefore goes beyond the scope of the Study, incorporating further reflections and discussions with scholars and experts that occurred after the Study was published. In Sect. 2 we outline the methodology of the Study. In Sect. 3 we synthesize, according to thematic headings, the main empirical findings of the Study and comment on whether they are expected or unexpected in the light of existing legal, management and economics literature on trade secrets. Finally, in Sect. 4, we examine the implications of these findings for legal policy in relation to EU trade secrets law.

The article argues for a cautious approach when it comes to reform of EU trade secrets law, suggesting minor adjustments to the recitals of the TSD and instead preferring to wait for judicial guidance to emerge from national courts and the CJEU. In the meantime, however, there is interpretative guidance and practical help that the European Commission can offer, along with monitoring and revisiting the relationship of trade secrets law to other areas of law, such as contract, copyright and the *sui generis* database right.

## Methodology

The key research question underpinning the Study was: to what extent can the legal protection of trade secrets help in creating a safe environment for business-to-business (B2B) data sharing? In seeking to answer this question a mixed methods approach was used by the research team.^22^ The Study conducted a literature review (which dealt with the management, economics and legal literature)^23^ and used quantitative and qualitative empirical approaches. The focus for the empirical research comprised four sectors^24^ – automotive, pharma/life sciences, energy/utilities and financial services. These sectors were chosen to reflect a breadth of business activity (including services vs. product related) and an expected growing significance of data sharing. A fifth sector (“Other”) was added, though, to account for respondents who would not consider themselves as part of the aforementioned four sectors.

An on-line survey, using a standardized questionnaire, was conducted between September 2021 and March 2022. The survey was deployed using a variety of channels, including social media, contacting European industry associations to share the survey with their members, an active search of individual experts and utilizing the professional networks of the researchers. There were 84 responses received: 40% of the respondents were large enterprises with 250 or more employees. The second largest share was comprised of research organisations,^25^ which make up 17% of the sample. Business associations accounted for 11% of responses. Only a few answers came from consultants (4%) and other types of organisations (2%, which are NGOs). In terms of the sectoral breakdown of respondents, the largest response rate came from the automotive industry (32.1%), followed by firms in health and life sciences (25%). Responses from the utility/energy (14.3%) and financial services sectors (11.9%) were low. In the “other sector” category was 35.7% of respondents, and these respondents came from chemistry, ICT, mechanical engineering, steel making, and semiconductor sectors, amongst others. Another factor to consider was that there was a tendency towards German-speaking countries (which made up 49% of the responses). This might be explained by the sampling procedure that favoured, to an extent, firms in the network of the research team. However, it must also be emphasised that Germany is the largest economy in Europe and accounts for around a third of patent applicants at the European Patent Office, for example.

In addition to survey evidence, 51 interviews were conducted between April 2021 and March 2022 with experts in trade secrets and/or data sharing, using a semi-structured interview guideline.^26^ From these interviews, and based on additional documentary analysis, 13 case studies were developed.^27^ A further source of evidence was the validation and dissemination workshop for the Study, which involved various stakeholders in the trade secrets and data sharing sphere.

The originality of the Study lies in the fact that it explores, from an empirical perspective, the *connection* between EU trade secret protection and shared confidential and commercially valuable (“CCV”) data.^28^ While there have been studies that analyse the motives and barriers for sharing of data, they have not focussed on the role of EU trade secrets law.^29^ Another study, from 2018,^30^ which deals with data access and control in the era of connected devices, considers the role of trade secrets protection, however, this is solely from a legal doctrinal perspective. Thus, the empirical focus on trade secrets and CCV data sharing that is featured in the Study is an important and valuable contribution to the existing literature. The contribution of this article is to extract and analyse, thematically, the main empirical findings of the Study and consider their implications for future EU legal policymaking.

## Main Empirical Findings

The empirical data are discussed in detail in the Study.^31^ The purpose of this section is to highlight the main empirical findings according to thematic areas and to comment on whether these were surprising or not, in the light of existing legal, management and economics literature. The key themes, important from the perspective of trade secret protection, which emerge from the survey and interview data may be classified under five headings: (i) data sharing practices; (ii) legal and practical mechanisms to protect shared data; (iii) motives for relying on trade secret protection to protect data; (iv) understandings of trade secret protection; and (v) barriers to relying on trade secret protection.

### Data Sharing Practices (Independent of Trade Secrets Protection)

The first set of empirical findings relate to data sharing practices independent of the role played by trade secrets protection. The Study found that sharing of CCV data is relevant to businesses, and is particularly pronounced in the automotive, life sciences and health industries.^32^ As well, respondents anticipated an increased relevance of sharing CCV data in future.^33^ The Study also found that classic data (such as know-how^34^) was more of a use case for data sharing than novel types of data (e.g. sensor generated data).^35^ In relation to novel types of data, the more relevant and valuable are processed, aggregated and structured data, compared with raw data.^36^ Further, the major barriers for firms sharing CCV data include the risk of losing competitive edge and a risk of losing control over data.^37^

These findings are not surprising. As data has become more important in the economy, so has interest in new ways of doing research and development, such as via open innovation.^38^ Open innovation, defined by knowledge flowing across organisational boundaries, even within the company, supports innovation,^39^ and is associated with higher levels of innovation and improved business performance.^40^ Thus, it is expected that respondents indicated the present *and* increased future relevance of sharing CCV data. In terms of the risks associated with sharing CCV data, this is also unsurprising considering Arrow’s (information) paradox.^41^ Arrow’s (information) paradox may be explained thus: to capture value from data, companies share data, often with collaborators and customers, but in doing so, companies must reveal their data for the other party to understand what they are purchasing. The very act of revealing reduces its value, as the other party now has sufficient information to have less need of the data. Finally, the fact that there is not yet a propensity of novel types of data being shared and that the least valuable type of data is raw data also accords with the literature.^42^

### Legal and Practical Mechanisms to Protect Shared Data

The next set of empirical findings relate to the legal and practical mechanisms that firms rely upon when sharing CCV data. A clear result is that, for respondents, contracts are the most important means to protect CCV data.^43^ This is followed by intellectual property (“IP”) protection (defined to include patents, copyright and the *sui generis* database right) and information technology/cybersecurity measures.^44^

The centrality of contracts to data sharing, followed by IP protection and technological measures, is a not unexpected result.^45^ Contracts are flexible, bilateral tools that can be used to facilitate data sharing^46^ and invariably, in the context of data sharing, include non-disclosure obligations.^47^ Meanwhile, technological measures are practical ways of controlling access to data, and these can include passwords, access codes, security questions or encryption.^48^ IP protection should also help address Arrow’s paradox because – in contrast to trade secrets protection – it offers exclusive property rights. However, it is surprising that respondents considered IP to be suitable for protecting CCV data. The IP schemes relevant to protection of data would be copyright and *sui generis* database right, yet these offer either a limited or uncertain scope of protection.

In relation to copyright, ideas, information and data per se are not protected,^49^ rather it is the creative expression of an author that is protected.^50^ The *sui generis* database right protects collections of data (as opposed to individual data).^51^ Moreover, it is contingent on substantial investment in collecting, rather than generating, data.^52^ This distinction is not straightforward to apply,^53^ although it has been suggested that the “substantial” threshold is an easy one to satisfy.^54^ Further, the line between generating and obtaining is a difficult one to draw in the case of connected devices and, while there has been some jurisprudence at Member State level to suggest that datasets of machine generated data would be protected,^55^ the CJEU has yet to decide this issue.^56^ This might explain why, in one interview, the opinion was expressed that the database right is relatively limited and should be clarified or broadened to include investment in the generation of data (including machine-generated data).^57^

### Motives for Relying on Trade Secret Protection

The empirical data also provide a window into why firms rely on trade secrets protection. The most important motive to use trade secrets protection for shared CCV data is to prevent misappropriation by third parties.^58^ Closely linked to this is the reason of having an additional safety net, should protection via contract fail.^59^ Thus, it is difficult to conclude that trade secrets protection has encouraged firms to share data. However, it does seem that the TSD has had some impact on firms’ activities, in so far as businesses have been prompted to review their legal, technical and organizational measures.^60^ For example, one of our interviewees (a large manufacturer of machines used in factories) reported that after the adoption of the TSD it trained its staff to classify information as for the public, for internal use only, and as confidential information only to be seen by select persons within the company.^61^ Training such as this would be relevant to identifying which CCV data is secret or not and help show that reasonable steps are taken for preserving secrecy.

Insofar as the empirical data points to reliance on trade secrets protection to prevent misappropriation of CCV data, this makes complete sense in light of the TSD, given that the directive requires harmonized protection of trade secrets via misappropriation, which it defines in Art. 4 as encompassing unlawful acquisition, use or disclosure of trade secrets and commercially dealing in infringing goods. Perhaps surprising, however, is that contracts are still seen as the primary mechanism for CCV data sharing while trade secrets protection is viewed as a “safety net”. The reason this is unexpected is because contractual regulation is bilateral in nature and offers only *in personam* protection, i.e. it does not extend to third parties. Whereas, EU trade secrets protection, even though it does not offer exclusive property rights,^62^ can extend to third parties. This is indicated by the scope of unlawful acquisition, use or disclosure in Art. 4 of the TSD. In the case of unlawful acquisition, this can reach third parties because it covers acquisition without consent of the trade secret holder by unauthorized access to, or appropriation of, or copying of any items from which the trade secret can be deduced. In relation to unlawful use or disclosure, this can be triggered by use or disclosure consequent upon having acquired the trade secret unlawfully. Moreover, Art. 4(2) TSD makes clear that third parties can be liable where they had actual or constructive knowledge that the trade secret had been obtained directly or indirectly from another person who was using or disclosing the trade secret unlawfully.

Finally, the fact that some (but not all) firms have instituted new protocols or training in response to the TSD is not unexpected. After all, the TSD is newly harmonized EU law and one that makes protection contingent on the existence of a trade secret, which in turn requires, *inter alia,* “reasonable steps” for preserving the secrecy of the information. Thus, one might expect firms to consider how best to comply with the regulatory change, for example, by assessing what mechanisms they have in place for maintaining the secrecy of their CCV data and to revise these where necessary. On the other hand, incorporating regulatory change is often complex for firms to grasp^63^ and so this is likely to account for the fact that only *some* of our respondent firms had changed their internal processes in response to the TSD.As Dr Freij points out (more generally, rather than in relation to trade secrets law), there is a research gap in “what firms do to manage implementation of new requirements after a change has been introduced”.^64^ Our empirical data hints at what firms have done, but a more thorough investigation into how the requirements of the TSD have been incorporated into firms behaviour could be pursued in future.

### Understandings of Trade Secrets Protection

The empirical data also reveal interesting results about the level of familiarity with, and understanding of, trade secrets protection. Amongst respondents there were mixed levels of familiarity with trade secrets, with the health and life sciences sector having the greatest familiarity.^65^ A clear point of uncertainty that emerges from the data relates to the meaning of “trade secret” and what might qualify as a “trade secret”.^66^ This uncertainty was said to be compounded by a lack of developed jurisprudence relating to the TSD.^67^ Despite varying levels of awareness of trade secrets, the data also indicates that this type of protection is frequently claimed.^68^

The lack of understanding of what constitutes a “trade secret” is both surprising and unsurprising. It is surprising insofar as the definition of “trade secret” is not new. Article 2(1) TSD is comparable to Art. 39 of the Agreement on Trade Related Aspects of Intellectual Property Rights (“TRIPS”), which has existed in international law since 1994. Further, Art. 2(1) TSD broadly reflects a “recurrence of certain common requirements” that existed in Member States laws prior to harmonisation.^69^ Moreover, there is developed, comparative jurisprudence of what constitutes a “trade secret” in the United States^70^ and Japan,^71^ where very similar definitions of “trade secret” are in operation.

The response is unsurprising, however, for two reasons. The first is that EU industry participants may not feel confident in assuming that the CJEU or national courts in Member States will take an approach similar to that which was taken previously in Member States, or which is taken in other jurisdictions. Second, comparative jurisprudence tends to focus on know-how and information generally and has not yet grappled with the complexities of how trade secrets law applies to the data economy. The applicability of trade secrets to the data economy is still relatively untested and unexplored in litigation, although it is an emerging area of scholarship.^72^

It is also unsurprising that there is limited European jurisprudence on the meaning of “trade secret”. The TSD was adopted in 2016 and the deadline for implementation was 9 June 2018. Therefore, we are looking at a relatively recently implemented law and one whose operation has occurred through the tumult of the COVID-19 pandemic. Moreover, trade secrets tend to be less frequently litigated than IP rights (such as patents or trade marks)^73^ and references on the TSD are likely to take some time to appear before the CJEU.^74^ Finally, while there is emerging jurisprudence from national courts in the EU, this has not yet tackled the specifics of the data economy.^75^

Finally, it makes sense that despite the uncertainty about what constitutes a trade secret, this type of protection is still claimed by organisations. This is because the definition of “trade secret” is broad: as indicated by recital 14 TSD, it includes technical information, know-how and business information. Provided the elements of secrecy, commercial value and reasonable steps are met, then there is nothing that *prima facie* precludes data (in the semantic sense) from being protected.^76^ Moreover, trade secrets protection is an unregistered right and, as such, there is no formal process for obtaining protection (as compared with patents or trade marks). As such, there is limited risk in asserting trade secrets protection, unless such an assertion is made in bad faith as part of litigation,^77^ and whether there is a protected trade secret will only be fully tested through litigation, in much the same way as we see in copyright law when it comes to whether there is a protected work.

### Barriers to Relying on Trade Secrets Protection

The final theme to emerge from the empirical data relates to barriers to firms relying on trade secret protection when it comes to data sharing. The major, reported barriers relate to enforcement; specifically, the difficulty of tracking or controlling the use of shared CCV data; the difficulty of assessing whether a trade secret has been misappropriated; and unclearness about whether legal enforcement of trade secrets is efficiently and effectively possible.^78^

In addition, respondents expressed concern about cross-border sharing of CCV data with China and the United States. This is attributed to inadequate protection of trade secrets and difficulties of enforcement.^79^ Another challenge that was raised is when CCV data is carried over by former employees to a new employer.^80^ The difficulties are identifying such instances and ascertaining the appropriate actions to be taken in response.

The findings on barriers to relying on trade secret protection have surprising and unsurprising elements. The surprising aspect of these results relates to cross-border sharing with the United States, in particular the perception that there is inadequate trade secret protection and difficulties of enforcement. This was an unexpected finding insofar as one can point to the United States as having very developed state and federal laws governing trade secrets. At state level, there is the Uniform Trade Secrets Act, which has been adopted by 47 states and forms the backbone of trade secrets law in the United States.^81^ In addition, there is federal trade secrets law in the form of the Economic Espionage Act 1996,^82^ which was amended in 2016^83^ to include civil redress alongside the existing criminal provisions.^84^

As well, it is surprising that there are concerns about the effectiveness of legal enforcement in the EU, given that a central plank of the TSD was to provide a harmonized, effective framework for enforcement^85^ that, in key (but not all) respects, mirrors that available in the IP Enforcement Directive.^86^ The TSD requires Member States to provide for a significant array of civil remedies, including interim measures (Art. 9), final measures (Art. 10), damages (Art. 13) and publication of judicial decisions (Art. 14). Given these are comparable in several respects to the Enforcement Directive^87^ one might have expected more confidence in the enforcement mechanisms.

There are four reasons that might account for the lack of confidence. First, to the extent that there are similarities between the TSD and Enforcement Directive, there has been only a small body of CJEU jurisprudence on the latter^88^ and so only a limited amount of judicial guidance has emerged at this stage that can be “transplanted” to the TSD. In any case, it may be questionable whether it is appropriate to transplant that jurisprudence because the Enforcement Directive and TSD are separate instruments and, where there is overlap, the TSD takes precedence as *lex specialis.*^89^ Second, to the extent that there are variations between the Enforcement Directive and TSD,^90^ the CJEU has not explicated those features unique to the TSD.^91^ For example, there is not yet an interpretation of the scope of who is the “trade secret holder”, which determines who has standing to sue, or about the factors relevant to proportionate remedies. Third, for some jurisdictions, the gap between previous law and implemented EU law is rather significant.^92^ In such jurisdictions, there is bound to be less awareness and familiarity with how enforcement will occur for trade secrets. Whereas, for those jurisdictions whose enforcement measures were already largely compliant, this is less likely to be the case.^93^ Finally, there is the fact of variation in national implementation of the enforcement measures. For example, there has been mixed implementation of Art. 9 of the TSD, which relates to preservation of confidentiality of trade secrets during litigation. To illustrate, the Czech Republic did *not* implement Art. 9 when implementing the TSD through Act No. 286/2018 because it relied on existing national rules that allow a judge to preclude the public from proceedings and to instruct persons to maintain the confidentiality of all trade secrets which they have heard during proceedings.^94^ However, this leaves open the question of access to, and use of, court documents that contain trade secrets, which is covered by Art. 9 of the TSD. By way of contrast, in the United Kingdom (which implemented the TSD before Brexit), Art. 9 of the TSD was fully implemented, even though English procedural law already provided adequate protection.^95^

The other concerns about enforcement reflected in the empirical data are less surprising. In relation to fears of inadequate protection and enforcement in China, this has some basis. As Jyh-An Lee, Jingwen Liu and Haifeng Huang have discussed,^96^ despite China bolstering its trade secret protection through amending its laws,^97^ this has not yet translated into more effective enforcement.^98^ The authors conducted an empirical study of trade secrets litigation in China from the period 2010–2020 and found that the win rate of claimants has been relatively low, with unsatisfactory damages awards and only a small proportion of cases involving foreign claimants. However, the authors also point out that there have been amendments to the procedural law in 2019 that should assist foreign claimants in future years.^99^ Therefore, we might expect greater confidence in data sharing with China in future.^100^

Finally, the other concerns raised about enforcement relate to legitimate and predictable practical difficulties – such as tracking or controlling the use of the shared CCV data; the difficulty of identifying when a trade secret has been misappropriated, including when it has been used to produce infringing goods; and employee transfer or leakage of trade secrets. Here, the mechanisms for dealing with these concerns are more likely to be managerial or organizational, rather than legal.^101^ Thus, the relevant issues regarding enforcement are likely to relate to technical and managerial ways of organizing and tracking data usage (and thus misuse), along with managerial improvements (such as education, training and clear data governance structures) to help minimize employee leakage.

## Implications for EU Trade Secrets Policy

In this section, we consider what lessons should be taken from our empirical findings when it comes to future EU policymaking for trade secrets protection. In response to the empirical findings, our recommendations relate to three areas: (i) clarification of the definition of trade secret; (ii) complementarity between trade secrets and other protection regimes; and (iii) effective legal enforcement. We argue that, while there are some minor, legislative improvements that could be initiated in relation to trade secrets, for the most part, it is a matter of preserving the status quo and allowing for jurisprudence to develop. Alongside this, information gathering about implementation of the TSD and workshops to increase knowledge and awareness of best practices would be helpful. It will also be important to ensure that the flexibility trade secrets protection allows when it comes to data sharing is not undermined by complementary forms of protection, such as contract, copyright and the *sui generis* database right. To that end, there needs to be serious consideration of abolition or reform of the database right.

### Clarification of the Definition of Trade Secret

Respondents across all industries indicated uncertainty about what would qualify as a trade secret in the data economy. This raises the question of whether to modify the legal definition of trade secret, in order to provide greater clarity about its application to data. Our answer is that it would not make sense to amend the definition, but that it might be useful to indicate some features of how the definition applies in the data economy using recitals or soft law guidance.

First, let us consider the existing definition in Art. 2(1) of the TSD, which states that, to qualify as a trade secret, information must meet the following requirements:

- it is secret in the sense that it is not, as a body or in the precise configuration and assembly of its components generally known among or readily accessible to persons within the circles that normally deal with the kind of information in question;
- it has commercial value because it is secret;
- it has been subject to reasonable steps under the circumstances, by the person lawfully in control of the information, to keep it secret.

It is clear, by reference to “information” that protection is at the semantic and not syntactic level^102^ and that, according to recital 14 TSD, the type of information that can be protected is broad. What our respondents struggled with, it seems, are the subsequent questions of whether the information has “commercial value” and whether there have been “reasonable steps” taken to protect secrecy. However, before turning to “commercial value” and “reasonable steps”, we briefly deal with the first requirement – i.e. “secrecy” because “secrecy” is the link between all three requirements.

#### Secrecy

The first thing to note is that information does not have to be secret in an absolute sense – relative secrecy will suffice. This is because Art. 2(1)(a) TSD refers to where information is not “generally known” or “readily accessible” to persons within the relevant circles (i.e. “persons within the circles that normally deal with the kind of information in question”). When considering the data economy, it therefore will be important to distinguish between information which is secret and that which is not. In relation to this, it is worthwhile keeping in mind that data generated from connected devices or data drawn from public sources is unlikely to satisfy the “secrecy” requirement. Therefore, while data may be valuable, this value may not (as discussed below) always arise because of the status of the information as “secret”.^103^ Moreover, this requirement for protection places a natural restriction on how much sharing of data is possible – too much sharing and eventually the data will become generally known or readily accessible, although what counts as “oversharing” will depend on the context (the circles normally dealing with that type of information).^104^

#### Commercial Value

Article 2(1)(b) of the TSD requires information to have “commercial value because it is secret”. Recital 14 of the TSD elaborates upon the meaning of “commercial value” and indicates that it may be “actual” or “potential”. Further, that:

know-how or information should be considered to have a commercial value, for example, where its unlawful acquisition, use or disclosure is likely to harm the interests of the person lawfully controlling it, in that it undermines that person’s scientific and technical potential, business or financial interests, strategic positions or ability to compete.

Recital 14 indicates that “value” may be assessed by the harm caused by trade secret misappropriation, where harm is conceptualised broadly as undermining various interests – whether they be technical, business, financial, or the ability to compete. In other words, the example is framed as *if* there was misappropriation (i.e. acquisition, use or disclosure of this information without permission), would the person lawfully controlling the trade secret be in a less competitive position, or lose money, custom, goodwill, etc. To put the question in its positive sense, it requires asking whether the information provides an *advantage* vis-à-vis its competitors. However, recital 14 overlooks a key element of the definition in Art. 2(1)(b) TSD, namely, that there must be commercial value *because* the information is secret as opposed to commercial value *per se.* Thus, an interpretation of commercial value must include not just the competitive advantage bestowed by the information (or the harm caused if it were misappropriated), but the fact that this advantage (or harm) arises *because* the information is secret.

If we turn to consider how “commercial value” relates to the data economy it seems unlikely that individual data will satisfy this requirement.^105^ On its own, individual data, such as a particular measurement or reading of a connected device relating to fitness, health, utilities, or cars, is not useful or meaningful in isolation, because the sensors on interconnected devices typically produce data that involve little semantic information. Further, the purpose of the TSD is to stimulate innovation and knowledge sharing and it is hard to imagine how individual or isolated data would contribute to that aim. Rather, it is only when individual data are combined into individual-level datasets (e.g., all data generated by a particular connected device) or aggregated datasets (all data generated by a multiple of connected devices) that such value may arise.^106^

In the case of individual level datasets generated from connected devices, access to such information by competitors does not necessarily destroy the competitive advantage of the manufacturer of the device. The exception is where the data relates to the technical functioning of the device and helps the manufacturer to improve the device and provide maintenance services (i.e. when the raw data becomes derived or inferred data).^107^

In the case of aggregated datasets, there are well-developed markets for non-personal data, relating, for example, to financial or commodities markets, credit scoring, weather, car matriculation data and geo-location data.^108^ Where specific markets exist for such diverse data, it may be possible to show that unlawful acquisition, use or disclosure of aggregated datasets undermines the trade secret holder’s business or financial interests, or its ability to compete. Even where markets for data do not yet exist, potential commercial value might nevertheless be established. However, it is important to ensure that such information has secrecy and commercial value *because of that secrecy*. It may be that data generated from connected devices (such as smart meters that track energy consumption), or gleaned from public sources (e.g. public records to ascertain information about bankruptcy, judgment debts or tax liens or social media in relation to credit scoring) lacks secrecy,^109^ and the aggregated version of this type of data will not change this status. Thus, while the data may have commercial value, this is not *because* of the secrecy of that information, as required by Art. 2(1) of the TSD.

Another consideration is whether datasets used to train AI may be protected as trade secrets. To the extent that much of the data is drawn from public sources, this is unlikely to be the case.^110^ Further, in instances where there is widespread availability of datasets, the secrecy requirement will not be met.^111^ To the extent that there is investment in “labelling” the training data for supervised learning, the dataset is more likely to reach the level of commercial value.^112^ But this does not mean that there is commercial value due to secrecy. If anything, the commercial value (i.e. competitive advantage) arises because the data can now be more effectively used. The same goes for where the dataset has been “cleaned” of redundant data.

When it comes to what qualifies as a trade secret in the data economy, it seems clear that individual data and raw (or unprocessed), predominantly machine-generated data will not be protected. Individual and aggregated datasets, however, are less straightforward if they are inferred or derived data and protection will depend on whether the data within is drawn from publicly or widely available sources (and thus is not secret) or from restricted sources. Even if the information is secret, commercial value must be causally connected to secrecy, as opposed to the usefulness of the data. These assessments of secrecy and commercial value will be context specific and difficult to set out as legislative rules. Thus, it does not seem wise to amend the definition of trade secret to try and reflect these different scenarios. Instead, it is preferable that any uncertainties are resolved through judicial interpretation. That said, it might be useful to provide guidance on the scope of the definition through the recitals to the TSD. Here, at the very least, a statement could be inserted that “individual data, raw (or unprocessed) data will not be protected” and recital 14 of the TSD could emphasise that commercial value must be due to secrecy.^113^

One legislative amendment that would be useful to make relates to the proposed Data Act^114^ and its interface with trade secrets. The proposed Data Act creates mandatory data sharing obligations in particular instances.^115^ According to recital 14 of the proposed Data Act, it will only apply to raw data generated or collected by connected devices, and will *not* apply to derived or inferred data.^116^ However, as discussed above, raw data from connected devices is unlikely to qualify as a trade secret either because it lacks semantic meaning, or secrecy (where it is exchanged on large data sharing platforms) or commercial value due to secrecy*.*^117^ Whereas, it is only when the raw data is processed to produce derived or inferred data, or aggregated into larger datasets, that commercial value will occur and, even in those instances, the competitive advantage must arise from the *secrecy* of the data. Thus, it appears that the data access obligations in the proposed Data Act would *not* clash with the trade secrets interests of data holders. If this is the case, then it is hard to see what role certain provisions – such as Arts. 4(3), 5(8), 17(2)(c) and 19(1) of the proposed Data Act – would have to play. Some commentators have remarked that the fact that the proposed Data Act is limited to raw data of the user (even if this can be a mixture of personal and non-personal data, and dynamic in nature) is highly problematic to achieving its aims.^118^ As such, it is recommended that the proposed Data Act be amended to include inferred and derived data, and even the aggregated dataset of multiple users.^119^ If this were the case then the purpose of the proposed Data Act would be better fulfilled, including the efficacy of Arts. 4(3), 5(8), 17(2)(c) and 19(1) which govern the interface with trade secrets. In this situation, it would be helpful also if, in the recitals to the proposed Data Act, it is clarified that “individual data, raw (or unprocessed) data will not be protected be as a trade secret”.

#### Reasonable Steps

The empirical data also raises the question of whether greater clarity can be provided on the requirement of “reasonable steps” to maintain secrecy. In determining what constitutes “reasonable steps”, there are questions about whether the assessment will be subjective, according to the circumstances of the business involved and the cost of those measures to that business, or whether it will be objective, measured by the usual protective measures that are adopted in the sector. There is only limited European jurisprudence so far, at the Member State level.^120^ More extensive case law on this topic exists in the United States, and U.S. commentators have indicated that the rationale behind this requirement is to give notice to third parties that the information is subject to legal protection.^121^ “Reasonable efforts”, as it is known in U.S. trade secrets law, requires a “highly factual and contextual analysis” and is treated as a question of fact.^122^ Reasonable measures do not require absolute secrecy, but relative secrecy, and there is a weighing up of the nature and value of the putative trade secrets and the cost of precautions to the putative trade secret holder. This suggests that the greater the value of the trade secret, the higher the standard of “reasonable measures”. In short, U.S. trade secrets law takes a relative, contextual and subjective approach to “reasonable measures” – i.e. analysing the type of trade secret in the context of the trade secret holder’s business.^123^

It is likely that national courts in EU Member States and the CJEU will adopt a similar approach to that in the U.S.,^124^ although there may still be a low, objective threshold that needs to be met, regardless of the type of business. For example, if a business decides to share data, a baseline “reasonable step” could be to use a non-disclosure agreement or non-disclosure obligations and to include a term requiring the licensee to take reasonable steps to ensure the information remains secret. In the case of digitally stored data, a minimum reasonable step could be to use technological protection measures to control access to that data. In anticipation of judicial clarification of “reasonable steps”, are there legislative amendments that should be made to provide more guidance to industry? We would advocate against prescribing in the statutory definition of “trade secret” what constitutes reasonable steps. This is because “reasonable steps” is a flexible standard that can be adjusted to a wide variety of contexts. To start articulating what constitutes “reasonable steps” would undermine this flexibility.

However, there is useful guidance that can be gleaned from U.S. case law about the types of measures that may be evidence of reasonable efforts. These are both internal and external to the organisation, such as: (i) use of non-disclosure or confidentiality agreements; (ii) restricting access to information; (iii) measures taken in relation to employees and ex-employees (e.g. exit interviews and terminating access to information systems once left); (iv) technological security measures; (v) physical security measures; and (vi) identifying and labelling information as confidential or trade secrets.^125^ Also, the size of the organisation may impact what constitutes “reasonable steps”^126^ and its level of sophistication may affect whether such steps are taken.^127^ While there is evidence to suggest that industry is already taking many of these steps, this is not happening across the board. Therefore, as opposed to trying to crystallise “reasonable steps” in the legislative definition of “trade secret” in the TSD, it is suggested that a more practical approach is taken. For example, the European Commission, after holding relevant stakeholder meetings, could issue guidance (in the form of interpretative soft law^128^) about the range of “reasonable steps” that may be taken. Further, the European Commission might consider hosting specific workshops for stakeholders to encourage industry dialogue about the practices that are routinely adopted in relation to maintaining the secrecy of their data.

### Complementarity Between Trade Secrets and Other Protection Regimes

The empirical data revealed that contractual means are routinely used for protecting CCV data. For many industry participants, contractual measures are both essential and prevalent because they can be tailored to determine access and sharing and the obligations of how to handle data. A variety of contracts may be used, but what they have in common, it seems, are non-disclosure obligations in relation to CCV data. As well, our empirical findings show that IP rights, such as copyright and the *sui generis* database right, are seen as key legal tools for protection of CCV data. It is therefore important that complementarity between trade secrets and these different legal regimes of protection – contract, copyright and database right – is maintained.^129^ We explore below how this might be better achieved.

#### Contract

In relation to contracts, the TSD is unlikely to disrupt the influential role of contractual agreements when it comes to data management and sharing. This is for several reasons. First, the TSD assumes that the protection it creates is in addition to that available under contract law. While the TSD is agnostic about the legal means of implementation (provided it does not create a property right as per recital 16), contract law would not suffice fully to implement the obligations in the Directive. Therefore, it is clear that contract sits alongside the obligations in the TSD. Second, use of contractual measures, such as non-disclosure agreements, confidentiality obligations on employees, or confidentiality obligations in transfer agreements, will be crucial for helping to establish the “reasonable steps” requirement for protection as a trade secret under Art. 2(1) of the TSD. Third, contractual obligations are a key means for determining when there is unlawful acquisition, use or disclosure of a trade secret under Art. 4 of the TSD. As such, contractual protection reinforces elements of EU trade secrets law. There is a mutuality that seems positive, and which should be continued.

That said, there are two areas where complementarity is harder to preserve. The first is the extent to which contractual measures can legitimately undermine or circumscribe lawful acts under Art. 3 of the TSD. The second is where contract leads to “overclaiming” of trade secrets protection. This refers to where contract is used to claim protection for information as “trade secrets”, even though such information would not satisfy the legal definition “trade secret”.

Turning first to the issue of contractual override of lawful acts, this arises in the case of reverse engineering in Art. 3(1)(b) of the TSD. This provision states that trade secret acquisition

shall be considered lawful when the trade secret is obtained by … (b) observation, study, disassembly or testing of a product or object that has been made available to the public or that is lawfully in the possession of the acquirer of the information who is free from any legally valid duty to limit the acquisition of the trade secret.

In cases of lawfully in the possession of the acquirer of the information, there must be no legally valid duty to limit the acquisition of the trade secret. Recital 16 of the TSD elaborates on this requirement, indicating that: “Reverse engineering of a lawfully acquired product should be considered as a lawful means of acquiring information, *except when otherwise contractually agreed*. The freedom to enter into such contractual arrangements can, however, be limited by law.” (emphasis added)

Although recital 16 suggests that there could be contractual override of *all* lawful acquisition by reverse engineering, when read together with Art. 3(1)(b) it seems clear that this is directed to instances where a person is in lawful possession of a product. In other words, it appears that agreements to hire, rent or license products *could* contain a provision that precludes study or disassembly for the purposes of reverse engineering. However, it is possible for Member States to limit the freedom to enter into such contractual arrangements. The same does not appear to be the case for contracts of sale and, by implication, this suggests that it is not possible contractually to override reverse engineering of products purchased on the open market.^130^ To give an example, a purchaser of an autonomous vehicle could legitimately pull it apart to understand how the vehicle operates (in terms of physical and IT engineering) without this constituting unlawful acquisition of a trade secret. However, to the extent that an autonomous vehicle is rented or hired by a third-party organisation, the manufacturer of the vehicles could prohibit those third parties from any kind of disassembly or study of the vehicle that enables it to understand its functioning.

From a normative perspective, contractual restrictions on lawful acquisition by reverse engineering are problematic where the product is software,^131^ because this creates an apparent inconsistency with copyright law, which makes exceptions for reverse engineering and decompilation imperative as a matter of EU law*.*^132^ However, it can be argued that the Software Directive is *lex specialis* and so this potential conflict would not really arise.^133^ Alternatively, it can be argued that, to the extent that software includes CCV data, reverse engineering should also be imperative as a matter of trade secrets law, in order to avoid undermining the reverse engineering copyright exception.^134^ Another argument is that a person is not “free from any legally valid duty to limit the acquisition of the trade secret” (as stated in Art. 3(1)(b) of the TSD) where to do so would be contrary to the copyright rules for software (specifically, Art. 8 of the Software Directive which makes contractual override of reverse engineering or decompilation of software null and void).^135^ To the extent that there is contractual override of reverse engineering for non-software products, whether this is problematic is likely to depend on whether such prohibition will undermine the innovation and price competition flowing from reverse engineering.^136^

Our empirical findings did not suggest that contractual restrictions on reverse engineering of lawfully acquired products were regularly in use, or, if they were in use, were currently having a deleterious effect on access to, or sharing of, CCV data. Nevertheless, it would be advisable for the EU Commission to monitor this situation, particularly since Member States may take different approaches to whether contractual override of reverse engineering in the case of lawful possession of a product is permissible.

Another issue is the extent to which contract may contribute to “overclaiming” of trade secrets protection. To understand this, we must appreciate that those who factually have control over data can assert “ownership” of the data as a trade secret via contractual agreements. While there are objective requirements under Art. 2(1) of the TSD, these are not assessed *ex ante,* as occurs with registered IP rights, such as patents. Therefore, it is possible for a data holder to assert, in a transfer, licensing or non-disclosure agreement, that the data they control and are sharing is a “trade secret”, even where the data is not secret, lacks independent economic value or has not been subject to reasonable steps for protection. In other words, contract allows data that is factually secret – as opposed to a trade secret – to be preserved and monetised.

Several observations can be made about this tendency. The first is that the uncertainty about whether the objective criteria of “trade secret” are satisfied in the context of the data economy (in conjunction with the lack of *ex ante* assessment) contributes to the tendency to assert trade secret “ownership” of data in contractual arrangements. Second, to the extent that the data is *not* a trade secret, this will mean that “ownership” can only be effectively enforced between the contractual parties – it will not be possible to enforce the protection in the TSD against third parties. However, this fact may not preclude a data holder from asserting trade secrets protection, which can, ultimately, only be tested by litigation. This creates a risk of third parties being sued for trade secret misuse (even if the courts do not ultimately uphold the claim), which in turn may generate more conservative behaviour on the part of third parties when it comes to data sharing. Thus, it is important that the application of the TSD to the data economy is clarified. Judicial interpretation has the advantage of flexibility and a context-sensitive approach; however, it may take considerable time for jurisprudence to develop. Therefore, in light of the potential negative impacts of “overclaiming” trade secrets protection when it comes to the data economy, at the very least, it would be advisable to follow the recommendation made previously, of clarifying in the recitals to the TSD that trade secrets do not apply to individual data, or raw/unprocessed data.

The use of contract to regulate access to information – even where it may not satisfy the requirements of Art. 2(1) TSD – also means that the checks and balances of trade secret law, particularly in Arts. 3 and 5 – can be circumvented.^137^ More attention therefore needs to be paid to whether those checks and balances should be applicable to factually secret data that does not reach the threshold of “trade secret”. This is a policy issue that warrants further investigation by the European Commission.

#### Copyright

As was mentioned above, copyright is likely to have limited application when it comes to data because the focus of protection is creative expression – facts, ideas and information are not protected.^138^ It is worth, however, expanding on two aspects of copyright law: copyright databases and software.

According to EU copyright law, databases are protected by copyright if “the selection or arrangement of their contents, constitute the author’s own intellectual creation”.^139^ The protection does not extend to the contents themselves, but only their “selection or arrangement”. An author’s own intellectual creation in the context of databases refers to “free and creative choices” and stamping a “personal touch”^140^ and this “criterion is not satisfied when the setting up of the database is dictated by technical considerations, rules or constraints which leave no room for creative freedom”.^141^ Thus, it is fair to say that copyright protection of databases does not extend to underlying data or information. Moreover, protection would only arise where there are creative choices in the selection or arrangement of data. In the context of the data economy, it is unlikely that these requirements will be fulfilled.^142^ This is because the data collected is likely to be comprehensive, and so little “selection” will be involved, and the arrangement of the data will be frequently dictated by technical choices.^143^ Moreover, one could argue that with connected devices the data is computer generated rather than human generated and, as such, there is an absence of an author.^144^

Turning next to consider software, it is clear this type of subject matter is protected as a literary work.^145^ In EU copyright law, it is also evident that copyright does not extend to the ideas and principles underlying any element of a computer program.^146^ As such, it appears that software that may drive machine learning or other data analysis will be protected by copyright, but not the underlying data on which it operates. However, there is one potential problem, namely, whether copyright protection for application programming interfaces (APIs) “can cause and aggravate data lock-ins”.^147^ This risk arises because APIs are crucial for interoperability of data formats and thus, in turn, for data portability and, if this type of software is copyright protected, the owner could control its use.^148^ In the United States, the copyrightability of APIs was assumed by the Supreme Court in *Google LLC v. Oracle America*^149^ although the court went on to find that the defendant’s copying of the API was fair use.^150^ It is debatable whether APIs are protectable in the EU. There is no CJEU ruling squarely on the issue, the closest being *SAS Institute Inc v. World Programming Ltd.*^151^ While the Court in *SAS Institute* held that “neither the functionality of a computer program nor the programming language and the format of data files used in a computer program in order to exploit certain of its functions constitute a form of expression of that program”,^152^ it also indicated that this finding did not preclude the possibility that programming languages and data file formats might be protected as works under Directive 2001/29 if they resulted from an author’s own intellectual creation.^153^ However, it can be argued that the technological constraints that shape APIs mean that creative choices are unlikely to be present, such that copyright does not arise.^154^ Still, the issue has not been definitively resolved in the EU and so some doubt remains. Importantly, if APIs were held to be copyright protected in the EU, the existing interoperability exceptions in the Software Directive – i.e. reverse engineering and decompilation^155^ – would not suffice. Thus, it may be that the Software Directive needs future amendment in order to include a data interoperability exception.^156^

It is argued that the existing boundaries within copyright law – of non-protection of ideas and facts and protection only of creative expression and, in the case of copyright databases, creative selection or arrangement of contents – are appropriate because they allow for the “free flow of information”.^157^ As well, it would be contrary to the rationales of copyright law to extend protection beyond creative expression to ownership of data. This is because copyright is usually justified as either an incentive to invest in creative expression or as a reward for that expression because it reflects the author’s labour or personality.^158^ Moreover, to the extent that copyright in software could prove an obstacle to data interoperability, it is important to investigate whether a new or revised interoperability exception is needed.^159^

In summary, it is suggested that the current boundaries to copyright protection are important vis-à-vis the purpose of copyright, but also to ensuring that there is no problematic overlap with trade secrets protection.^160^ However, it will be important to monitor whether copyright protection of software unduly interferes with data interoperability.

#### Database Right

As was discussed above, the *sui generis* database right creates a property right in *collections* of data that are the result of substantial investment. The existence and scope of the database right has long been contentious^161^ and this continues to be the case when one considers the data economy.^162^ For example, the 2018 Final Report evaluating the Database Directive suggests this protection scheme is unsuitable and out-dated for a data-driven economy.^163^ The Final Report notes uncertainties regarding the applicability of the *sui generis* database right to machine or sensor generated data, the identification of the database maker, and whether new types of activities, such as web scraping, amount to infringement of the database right. It also considers – without firmly recommending – introducing a compulsory licensing system for sensor-produced databases.^164^ On this latter point, some scholars have advocated for compulsory licenses, in relation to sole source databases, including those that have not been published, on condition of fair and non-discriminatory remuneration.^165^ Others, however, are sceptical about the value of a compulsory licensing system for dealing with “data lock-ins that result from *de facto* data control” and call instead for a focus on data access rights that prevail over any database right.^166^

Some of the concerns about the breadth of database right protection, borne from earlier CJEU rulings,^167^ have been addressed by the ruling in *CV-Online Latvia v. Melons*.^168^ While the Court maintained a broad interpretation of “extraction” and “reutilisation”, it sought to balance the substantial investment of database makers and the legitimate interests of database users, such as to create innovative products, by stating that the extraction or re-utilisation must constitute “a risk to the possibility of redeeming that investment”.^169^ Meanwhile, the suggestion of data access rights that override the *sui generis* database right has been adopted in Art. 4(1) of the proposed Data Act, which places an obligation on a data holder to make available to the user the data generated by the user’s use of a product or related service (where this cannot be directly accessed by the user). Further, Art. 5(1) of the proposed Data Act obligates a data holder to make data generated by the use of a product or related service available to a third party acting on behalf of a user. Importantly, Art. 35 of the proposed Data Act states that, in order not to hinder Arts. 4 and 5, the *sui generis* database right “does not apply to databases containing data obtained from or generated by the use of a product or a related service”. This seems to be a *lex specialis* provision and one that has been welcomed, albeit with various suggestions for improving clarity.^170^

From the point of view of ensuring complementarity between trade secrets and database right protection in the data economy, it would be a pity to undermine the flexible, unfair competition type regime offered by trade secrets with a property-based regime that has the propensity to foster data lock-ins. Therefore, it is argued that the proposed Data Act adopts the correct structural approach of overriding the *sui generis* database right when it conflicts with mandatory data access and sharing obligations. However, it could be argued that reform should go further, and the *sui generis* database right should be repealed. This is because – according to European Commission studies – the right is of questionable value, with no evidence of positive impact since its introduction.^171^ In the absence of this more radical step, which admittedly might give rise to concerns about whether this is contrary to Art. 17(2) of the EU Charter of Fundamental Rights, it is worth considering legislative amendments to the Database Directive to clarify what sorts of investments are applicable (and non-applicable) to the database right^172^ and to codify infringement principles according to the CJEU ruling in *CV-Online.*^173^ As well, the exceptions to the database right should be further aligned with those in copyright law^174^ (which has been achieved to some extent via the Digital Single Market Directive)^175^ and a compulsory license provision should be introduced.^176^

### Effective Enforcement

As discussed above, concerns about whether there is an adequate legal framework for enforcement of trade secrets are generally misplaced, except in relation to data sharing in China (although this looks likely to improve). Therefore, it does not seem that legislative changes are needed when it comes to EU trade secrets enforcement. Rather, the challenges relate to awareness and understanding of the new enforcement framework in EU trade secrets law and practical difficulties associated with enforcement (e.g. identification of misappropriation).

A better understanding of the trade secrets enforcement framework will occur once national court and CJEU jurisprudence emerges, although this may take some time. In the interim, it would be helpful for the European Commission to undertake a systematic mapping of how the enforcement provisions of the TSD have been implemented in different Member States, in order to identify whether there is compliance, but also where there might be variation. In addition, educational or awareness raising workshops could be useful, particularly in those Member States where there have been significant changes to procedural law as a result of the TSD. On the practicalities of enforcement, it may be helpful to arrange best-practice workshops in order to encourage greater industry knowledge about the most effective measures to track and identify trade secret misuse, and to share ways in which to best manage employees in order to ensure effective data governance and avoid leakage.

In other words, our recommendation is that the concerns about legal enforcement of trade secrets require pragmatic rather than legal responses at this stage.

## Conclusion

The empirical data from our Study constitute an important and original contribution to the legal, management and economics literature on trade secrets and the data economy. This article has distilled that empirical data into the following thematic areas: CCV data sharing practices (independent of trade secrets protection); legal and practical mechanisms to protected shared data; motives for relying on trade secret protection; understandings of trade secret protection; and barriers to relying on trade secret protection. The major findings are first, independent of trade secrets protection, CCV data sharing is highly relevant to businesses but a major barrier to doing so is the fear of losing control over the data and a competitive edge. Second, the primary legal mechanism relied upon by businesses when sharing CCV data is contract law, followed by IP rights, such as copyright and database right. Trade secrets remain a relevant form of legal protection, primarily when contract fails or in the event of a misappropriation by third parties. Third, despite our respondents seeing the relevance of trade secrets, there is unfamiliarity with trade secrets law, in particular, uncertainty about the scope of the definition of “trade secrets”. Finally, the major barrier to relying on trade secrets was said to be difficulties of enforcement.

After reflecting on the empirical data, our recommendation for EU legal policymaking for trade secrets law is to adopt a cautious approach. In terms of legislative reforms of the TSD, we recommend a modest change, which is to clarify, in the recitals, that “individual data, raw (or unprocessed) data will not be protected”. In relation to “reasonable steps”, we recommend that soft law or guidance from the European Commission be utilised instead of any amendment to the definition of “trade secret”. With regards to other legislation, there are amendments that can be made. The proposed Data Act should clarify, in its recitals, that individual, raw or unprocessed data is not protected as a trade secret and that the scope of the Data Act *does* extend to inferred and derived data and the aggregated datasets of multiple users. When it comes to preserving complementarity between trade secrets and other legal regimes, it would be wise for the European Commission seriously to explore abolishing the *sui generis* database right or else to introduce express amendments to clarify the threshold and scope of protection, align exceptions more closely with copyright exceptions for databases and introduce a compulsory licence provision. In relation to complementarity with copyright law, this is largely fine, other than to monitor whether software copyright unduly interferes with data interoperability. Greater attention, however, should be paid to complementarity with contract law, in particular, whether there is a widespread practice of contractually overriding reverse engineering activities (especially for software products containing trade secrets) and considering to what extent it is legitimate to create factual exclusivity over data via contract that bypasses the limitations that exist in trade secrets law. Finally, when it comes to legal enforcement of trade secrets, we advocate for practical – rather than legal – steps being adopted for the time being, since the TSD introduced a robust enforcement framework that simply needs time to percolate through national laws and practice. Moreover, some of the enforcement concerns that were raised can only be addressed via managerial and organizational measures.

## Footnotes

1 European Commission (2020).

2 *See* Drexl et al. (2019).

3 Directive 96/9/EC of the European Parliament and of the Council of 11 March 1996 on the legal protection of databases OJ L 77, 27.3.1996, pp. 20–28 (Database Directive).

4 Directive (EU) 2019/790 of the European Parliament and of the Council of 17 April 2019 on copyright and related rights in the Digital Single Market and amending Directives 96/9/EC and 2001/29/EC OJ L 130, 17.5.2019, pp. 92–125 (DSM Directive).

5 Regulation (EU) 2016/679 of the European Parliament and of the Council of 27 April 2016 on the protection of natural persons with regard to the processing of personal data and on the free movement of such data, and repealing Directive 95/46/EC (General Data Protection Regulation) OJ L119, 4.5.2016, p. 1.

6 Regulation (EU) 2022/1925 of the European Parliament and of the Council of 14 September 2022 on contestable and fair markets in the digital sector and amending Directives (EU) 2019/1937 and (EU) 2020/1828 (Digital Markets Act) OJ L 265, 12.10.2022, pp. 1–66.

7 Regulation (EU) 2022/2065 of the European Parliament and of the Council of 19 October 2022 on a Single Market for Digital Services and amending Directive 2000/31/EC (Digital Services Act) OJ L 277, 27.10.2022, pp. 1–102, especially Art. 40.

8 Regulation (EU) 2022/868 of the European Parliament and of the Council of 30 May 2022 on European data governance and amending Regulation (EU) 2018/1724 (Data Governance Act) OJ L 152, 3.6.2022, pp. 1–44.

9 Data Act Proposal – *see* https://digital-strategy.ec.europa.eu/en/library/data-act-proposal-regulation-harmonised-rules-fair-access-and-use-data. Trilogue negotiations are ongoing and are unlikely to be finalised before Spring 2023.

10 Directive (EU) 2019/1024 of the European Parliament and of the Council of 20 June 2019 on open data and the re-use of public sector information OJ L 172, 26.6.2019, pp. 56–83.

11 Directive (EU) 2015/2366 of the European Parliament and of the Council of 25 November 2015 on payment services in the internal market, amending Directives 2002/65/EC, 2009/110/EC and 2013/36/EU and Regulation (EU) No. 1093/2010, and repealing Directive 2007/64/EC (Text with EEA relevance) OJ L 337, 23.12.2015, pp. 35–127*.*

12 De Jongh et al. (2018); technically, this is also compliant with Art. 39(3) TRIPS. *See* Directive 2011/83/EC of the European Parliament and of the Council of 6 November 2011 on the Community code relating to medicinal products for human use OJ L 311, 28.11.2011, p. 67.

13 Regulation (EC) No. 715/2007 of the European Parliament and of the Council of 20 June 2007 on type approval of motor vehicles with respect to emissions from light passenger and commercial vehicles (Euro 5 and Euro 6) and on access to vehicle repair and maintenance information (Text with EEA relevance) OJ L 171, 29.6.2007, pp. 1–16 and Regulation (EC) No. 595/2009 of the European Parliament and of the Council of 18 June 2009 on type-approval of motor vehicles and engines with respect to emissions from heavy duty vehicles (Euro VI) and on access to vehicle repair and maintenance information and amending Regulation (EC) No. 715/2007 and Directive 2007/46/EC and repealing Directives 80/1269/EEC, 2005/55/EC and 2005/78/EC (Text with EEA relevance) OJ L 188, 18.7.2009, pp. 1–13.

14 Regulation (EU) 2019/631 of the European Parliament and of the Council of 17 April 2019 setting CO2 emission performance standards for new passenger cars and for new light commercial vehicles, and repealing Regulations (EC) No. 443/2009 and (EU) No. 510/2011 (recast) OJ L 111 25.4.2019, p. 13.

15 *See* https://energy.ec.europa.eu/topics/energy-strategy/clean-energy-all-europeans-package_en for details.

16 [2016] OJ L157/1. Adopted 8 June 2016, with an implementation deadline for Member States of 9 June 2018. For an overview of the Directive and implementation in key member states *see* Schovsbo et al. (2020).

17 *See* recitals 2–4, 8 TSD.

18 Drexl (2018), p. 91.

19 Recital 2, TSD.

20 For an analysis, *see* Drexl (2018). *See also* Aplin (2017); and Nordberg (2020).

21 European Commission (2022).

22 For a more detailed discussion *see* Study, Section 2.

23 *See* Study, Section 3.

24 In defining the four industries, a rather open ecosystem/value chain approach was employed by which, for example, suppliers in different positions of the value chain for an industry were also considered to be part of that industry. That way we also tried to account for the changing and blurring “borders” of industries due to rapid technological change and business model innovation – an aspect particularly relevant for the subject matter scrutinised in the Study.

25 Comprising both universities and non-university organisations.

26 *See* Study, Section 3.2. The semi-structured interview guideline overlapped with the survey topics, insofar as it sought to explore the interviewees familiarity with the study topic, usage, motives and barriers to confidential and commercially valuable (“CCV”) data sharing, the types of data shared, the scenarios for data sharing, how data assets are identified, how CCV data is protected, the modes and conditions for data sharing, along with considering any international issues and whether breaches of agreements to share CCV data occur.

27 *See* Study, Appendix C.

28 Our focus was on data that is confidential and commercially valuable, as opposed to data generally. This was in order to more effectively explore the role of EU trade secrets law in influencing data sharing practices.

29 *See* European Commission (2021); and European Commission (2018b).

30 Drexl (2018).

31 Study, Section 4 and Annex C for case studies.

32 Study, pp. 44–45.

33 Study, p. 45.

34 *I.e*., company or employer know-how, as opposed to individual/employee know-how.

35 Study, pp. 39 and 51.

36 Study, pp. 39, 53, 54. *See also* Study, Appendix C, Case Study 7.

37 Study, pp. 57–58. *See also* Study, Appendix C, Case Studies 1 and 2.

38 Bader (2006) and Chesbrough (2003).

39 Bader (2008) and King (2007).

40 Chesbrough (2017).

41 Arrow (1962).

42 *See* Coyle and Manley (2022), p. 20; Drexl, (2018), pp. 41–42; World Economic Forum (2021), pp. 8–9 (discussing the data value chain and how the value multiplies beyond the raw data stage).

43 Study, pp. 2, 40, 49, 56, 58, 67, 69. *See also* Study, Appendix C, Case Studies 1, 2, 4, 5, 7, 8, 10, 12 and 13.

44 Study, pp. 58–60. Although note that IP rights were less important to organisations in the financial sector: *see* Study, Appendix C, Case Studies 5 and 9.

45 Ziegler et al. (2013); and Bonakdar et al. (2017).

46 For example, the “assignment” of trade secrets regularly happens in practice via contract, although often part of a larger transaction involving several intellectual property rights: *see* standard forms and precedents in this area, as discussed in Cook and Horton (1998); Melville (2006).

47 *E.g.*, *see* Study, Appendix C, Case Studies 1, 2, 10, 11 and 13.

48 Such access controls (alongside copy-protection controls) have been utilised in relation to copyright works for decades and there are legal prohibitions on circumvention of technological protection measures applied to copyright works (but not to data). *E.g.*, *see* WIPO Copyright Treaty 1996, Art. 11, WIPO Performances and Phonograms Treaty 1996, Art. 18 and Information Society Directive, Art. 6.

49 In support of this *see*: WIPO Copyright Treaty 1996, Art. 2 and TRIPS Agreement, Art. 9(2), Directive 2009/24/EC on the legal protection of computer programs OJ L 111, 5.5.2009, pp. 16–22 (Software Directive), rec 11 and Art. 1(2), and *SAS Institute Inc v. World Programming Ltd* Case C-406/10, EU:C:2012:259, paras. 31–33.

50 A copyright work must be original in the sense of an author’s own intellectual creation: see *Infopaq International A/S v. Danske Dagblades Forening* Case C-5/08, EU:C:2009:465, para. 37, and *Football Association Premier League* C-403/08 and C-429/08, EU:C:2011:631, para. 97, which refers to creative choices that reflect an author’s personality: *Painer v. Standard Verlags GmbH* Case C-145/10, EU:C:2011:798, paras. 88–89; and the protection of the work extends to the elements which are the expression of the author’s own intellectual creation: *Infopaq,* para. 39.

51 Database Directive, Art. 1(2): defining “database” as a “collection of independent works, data or other materials arranged in a systematic or methodical way and individually accessible by electronic or other means”.

52 As stated by the CJEU in *British Horseracing Board Ltd v. William Hill Organization Ltd* Case C-203/02, EU:C:2004:695, paras. 31–32.

53 *See* Davison and Hugenholtz (2005), p. 115 and note that *Football Dataco v. Sportradar* [2013] EWCA Civ 27, para. 39 (Jacob LJ, with Lewison and Lloyd LJJ in agreement) held that a database of “live” information about what was occurring in football matches was not creating data, but was recording (and thus collecting) data.

54 Derclaye (2005), pp. 20–21; Leistner (2002), pp. 448–449.

55 Case 1 ZR 47/08 *Autobahnmaut* [2010].

56 Drexl (2018), pp. 71–73.

57 *See* Study, Appendix C, Case Study 8.

58 Study, pp. 67–68.

59 Study, pp. 67–68.

60 Study, p. 47. *See also* Study, Appendix C, Case Study 1.

61 Study, p. 40.

62 *See* recitals 1 and 10, TSD; Knaak et al. (2014), paras. 16–17.

63 *See* Freij (2017), pp. 15 and 17. Also noting at p. 22 that “Very few academic or practical studies have provided any information on what firms do to manage new requirements from regulatory change”. Dr Freij argues that regulatory change influences products, processes and technology.

64 *Ibid*, p. 22.

65 Study, pp. 62–64.

66 Study, pp. 70, 72. *See also* Study, Appendix C, Case Study 4.

67 Study, p. 69.

68 Study, pp. 65–66.

69 De Martinis et al. (2013), p. 5.

70 Analysing the lessons from U.S. trade secret law for the “reasonable steps” requirement of “trade secret” *see* Beale and Foulser McFarlane (2020). Analysing the similarities between the EU TSD and U.S. trade secret law *see* Sandeen (2017); and Wennakoski (2016). On U.S. trade secret law more generally *see* Milgrim and Bensen (2019); Pooley (2022); and Sandeen and Rowe (2022).

71 Suzuki (2021), ch 1. *See also* Ministry of Economy (2003).

72 *See*, *e.g.*, Drexl (2018), pp. 93–106; Leistner (2021a, b), pp. 232–235; Nordberg (2020); and Sandeen and Aplin (2022), ch. 24.

73 *E.g.*, *see* Willis Towers Watson, pp. 9–10, discussing IP litigation in the U.S. and noting that between 2012–2017 there was an average of 5,200 patent infringement cases filed in U.S. federal courts per year and between 2009 and 2016 there was an average of 3,900 trade mark cases filed in federal courts per year. Whereas, between 1994 and 2012, there was an average of 147 trade secret cases filed per year in federal court, although the numbers are increasing following the introduction of the Defend Trade Secrets Act 2016. We have not been able to find comparable data for the EU, but there is no reason to think that the broad trends would not be the same.

74 *See* Cook (2014), p. 57, who observes that references to the CJEU may be rare “given the long timescales involved in such references when ranged against the fleeting nature of much trade secrets protection, manifested in disputes that are usually resolved after applications for interim relief and which only rarely get as far as a full hearing on the merits.”

75 In the UK, which was obliged to implement the TSD before its departure from the EU, there has been consideration of national implementation of the TSD in *Shenzen Senior Technology Material Co Ltd v. Celgard, LLC* [2020] EWCA Civ 1293, para. 28 (Arnold LJ). *See also* De Vroey and Allaerts (2021); Germany (2021) 52(6) IIC 775 and Poland (2020) 51(9) IIC 1129.

76 Aplin (2017) and Drexl (2018), pp. 92–93 (referring to data collected through connected devices).

77 Art. 7(2) of TSD requires Member States to ensure that judicial authorities can apply appropriate measures where legal proceedings for trade secret misuse is “manifestly unfounded” and initiated “abusively or in bad faith”. Appropriate measures “include awarding damages to the respondent, imposing sanctions on the applicant or ordering the dissemination of information concerning a decision as referred to in Article 15”. *See also* recital 22 which indicates that such bad faith litigation may be for “the aim of unfairly delaying or restricting the respondent's access to the market or otherwise intimidating or harassing the respondent.”

78 Study, pp. 69 and 72, 73.

79 Study, pp. 60–61, reporting on 79% of respondents associating these problems with China and over 50% associating them with the United States.

80 Study, Case Study 7, Appendix C.

81 Sandeen and Rowe (2018).

82 *See* 18 U.S.C., chapter 90, § 1831, *et seq.*

83 Defend Trade Secrets Act 2016, Public Law 114–153, May 11, 2016.

84 For a discussion: *see e.g.*, Sandeen and Seaman (2017); Levine and Seaman (2018).

85 For discussion *see* Aplin (2014).

86 Directive 2004/48/EC on the enforcement of intellectual property rights, OJ L195/16, 2.6.2004.

87 For a detailed comparison *see* Riis (2020), ch. 12.

88 *E.g.*, *Liffers v. Producciones Mandarina SL,* Case C-99/15, ECLI:EU:C:2016:173 (on damages for moral prejudice); *Mircom International Content Management & Consulting (MICM) Ltd v. Telenet BVBA* Case C-597/19, EU:C:2021:492 (on persons entitled to remedies); *Cooperatieve Verenigin SNB-REACT UA v. Mehta* Case C-521/17, EU:C:2018:639 (whether collective body representing trade mark owners had legal standing under the Enforcement Directive); *Stowarzyszenie Olawska Telewizja Kablowa (OTK) v. Stowarzyszenie Filmowcow Polskich (SFP)* Case C-367/15, EU:C:2017:36 (on damages calculations).

89 Rec. 39, TSD; Riis (2020), p. 222.

90 Aplin (2014), pp. 276–277 explains they are comparable apart from three key differences, which relate to (i) the persons entitled to seek measures, procedures and remedies; (ii) the absence of remedies in the TSD for preserving evidence or for obtaining orders regarding the origin and distribution networks of infringing goods; or for obtaining interim or final injunctions against intermediaries whose services are used to infringe a trade secret; and (iii) the fact that TSD has explicit factors for the court to consider when determining proportionate remedies.

91 There has, however, been some scholarly attention: *e.g.*, *see* Mylly (2022).

92 Take the example of Finland which did not have provisions approximating Art. 9 of the TSD: *see* Schröder (2018).

93 An example here is the United Kingdom, which implemented the TSD before Brexit: *see* Aplin and Arnold (2020), ch. 5, esp. pp. 80–85.

94 *See* Chloupek (2019).

95 *See* Trade Secrets (Enforcement, etc.) Regulations 2018 SI 2018/597, which came into force on 9 June 2018, specifically regulation 10 and Aplin and Arnold (2020), p. 81.

96 Lee et al. (2022).

97 *See* Anti-Unfair Competition Law of the People’s Republic of China (2019 Amendment), discussed in Lee (2020), p.173.

98 This view is also supported by Wang and Chang (2019).

99 Lee et al. (2022), p. 774.

100 Although Wang (2023) suggests that there is still a need for improved legislation and judicial interpretation when it comes to trade secrets protection in China.

101 It is interesting to note that an issue that did not come up in the survey or interview data is the extent to which different Member States laws regulating employees/ex-employees complicates how organisations minimise and manage trade secret leakage by employees.

102 Drexl (2018), p. 92.

103 *Ibid*, p. 94; Sandeen and Aplin (2022).

104 Here it is interesting to note that in Study, Case Study 3, Appendix C, an OEM automotive supplier stated where its data is shared, it is no longer considered a trade secret.

105 *See* Aplin (2017) and Noto La Diega and Sappa (2020); Drexl (2018), p. 93.

106 *See* Noto La Diega and Sappa (2020); Drexl (2018), p. 93.

107 Drexl (2018), p. 94.

108 European Commission (2017), p. 13; Mayer-Schönberger and Cukier (2013), pp. 89–91.

109 Drexl (2018), p. 94; Sandeen and Aplin (2022).

110 Sandeen and Aplin (2022).

111 Peng et al. (2021) traces how two popular face and person recognition datasets (DukeMTMC and MS-Celeb-1M) remain widely available even after retraction by their originators, which they call “runaway data”.

112 Labelling means the training data is labelled as to what it represents, which allows the supervised learning model to determine whether its prediction was right or wrong: *see* Drexl et al. (2019).

113 On the role of recitals in EU law *see* Klimas and Vaiciukaite (2008).

114 *See* https://digital-strategy.ec.europa.eu/en/library/data-act-proposal-regulation-harmonised-rules-fair-access-and-use-data.

115 For a detailed evaluation *see* Drexl et al. (2022).

116 Present recital 14 of the proposed Data Act states: “Physical products that obtain, generate or collect, by means of their components, data concerning their performance, use or environment and that are able to communicate that data via a publicly available electronic communications service (often referred to as the Internet of things) should be covered by this Regulation … The data represent the digitalisation of user actions and events and should accordingly be accessible to the use, while information derived or inferred from this data, where lawfully held, should not be considered within scope of this Regulation”. Note that in the Draft Report by Committee on Industry, Research and Energy (2022), there are amendments proposed to rec 14. However, the changes extend to including raw data and “prepared data” (defined as “data cleaned and transformed for the purpose of making it useable prior to further processing and analysis”) but not derived or inferred data.

117 Drexl (2018), p. 94.

118 *See* Kerber (2022), pp. 11 and 12, referring to the covered data as too “narrow” to enable third parties “to offer additional services to the users like repair or predictive maintenance services on downstream or adjacent markets”.

119 *Ibid*, p. 12.

120 De Vroey and Allaerts (2021), p. 1394 briefly discuss a few cases decided before the TSD was implemented that could be relevant to reasonable steps. There has been limited discussion by European scholars, although *see* Mylly (2021), pp. 1329–1330.

121 Bone (2011), p. 59 and Sandeen and Rowe (2018), pp. 93–94.

122 Sandeen and Rowe (2018), p. 94 citing *Rockwell Graphic Sys Inc v. DEV Indus., Inc*., 925 F. 2d 174, 176–77 (7th Cir. 1991).

123 Sandeen and Rowe (2018), p. 100: “The inquiry necessarily varies in each case based on the costs of the protective measures relative to the risks of misappropriation and the attendant benefits of protecting the information”.

124 For example, Prof. Angsar Ohly discusses how “reasonableness” is a “flexible, malleable and relative concept” and how the German government has provided basic criteria of the absolute value of the trade secret, its relative value to the trade secret holder and the costs and availability of protection measures: *see* Ohly (2020), at p. 109.

125 *See* Sandeen and Rowe (2018), p. 101 and Beale and McFarlane (2020).

126 *See Puroon Inc. v. Midwest Photographic Res Ctr Inc.,* 2018 WL 5776334 (N.D. Ill. Nov 2, 2018) and *Elmer Miller Inc. v. Landis,* 253 Ill. App. 3d 129 (1st Dist. 1993).

127 Our interview with a U.S. legal expert suggested that U.S. companies either take a sophisticated approach to trade secrets, categorising their information and tailoring their protection measures accordingly; or they take a crude approach of lumping all information together and regularly using non-disclosure agreements in relation to sharing such information; or they take few measures.

128 It is appreciated that there is much contention over the influence and impact of EU soft law, which has been investigated at length, for example, in Eliantonio et al. (2020). *See also* Andone and Coman-Kund (2022).

129 Of course, other legal tools, such as tort law claims, may arise, but these did not emerge from our empirical data.

130 *See also* Ohly (2020), pp. 115–116.

131 Aplin (2013), pp. 32–33.

132 *See* Software Directive*,* Art. 8: “Any contractual provisions contrary to Article 6 or to the exceptions provided for in Article 5(2) and (3) shall be null and void”. *See also SAS Institute Inc v. World Programming Ltd* Case C-406/10, EU:C:2012:259, paras. 47–62 on the relationship between Arts. 5(3) and 8 of Council Directive 91/250/EEC of 14 May 1991 on the legal protection of computer programs OJ 1991 L 122, p. 42.

133 Noto La Diega (2018) para. 35 and Udsen et al. (2020) p. 35.

134 Aplin (2013), pp. 32–33.

135 Mylly (2021), pp. 1325–1326.

136 Aplin (2013), pp. 4–5, discussing the rationale for allowing reverse engineering.

137 In much the same way as occurred in Case C-30/14 *Ryanair Ltd v. PR Aviation BV,* ECLI:EU:C:2015:10 and critiqued by Borghi and Karapapa (2015).

138 *See supra* note 49 above. *See also* Drexl (2018), p. 32.

139 Art. 3(1) Database Directive.

140 *Football Dataco v. Yahoo! UK Ltd* Case C-604/10, EU:C:2012:115 (Third Chamber), para. 38.

141 *Ibid*, para. 39.

142 Drexl (2018), p. 86; and Leistner (2021a), p. 386.

143 The decision in *Funke Medien NRW GmbH v. Bundesrepublik Deutschland* C-469/17, ECLI:EU:C:2019:623, para. 24 would support this view too. Although the case did not concern a database, but rather military status reports, it does suggest the limited scope for intellectual creation in relation to informational works: here the CJEU indicated that purely informative documents would often by characterized by their technical function and thus struggle to meet the originality threshold.

144 Hugenholtz (2017), p. 85.

145 WIPO Copyright Treaty 1996, Art. 4; TRIPS, Art. 10(1) and Software Directive, Art. 1(1).

146 Software Directive, rec 11 and Art. 1(2), and *SAS Institute Inc v. World Programming Ltd* Case C-406/10, EU:C:2012:259, paras. 31–33.

147 Drexl (2018), p. 86.

148 *Ibid*.

149 *See Google LLC v. Oracle America, Inc* 141 S. Ct. 1183 (2021) No. 18-956, 2021 WL 1240906 (U.S. Apr. 5, 2021), 1197.

150 *Ibid,* 1200–1209.

151 Case C-406/10, EU:C:2012:259.

152 *Ibid*, para. 39.

153 *Ibid*, para. 45.

154 *See* Leistner (2021a), p. 385 who suggests APIs are unlikely to be protected as software copyright in the EU.

155 Software Directive, Arts. 5(3) and 6.

156 Drexl (2018), p. 87.

157 *Ibid*, p. 86.

158 This is an oversimplified statement of the various copyright justifications and there is a vast literature on this topic: for a synthesis *see* Spence (2002) and Aplin (2005). It is also important to note that there are less mainstream justifications for copyright, such as that in Drassinower (2015) (arguing that a copyright work is a communicative act and infringement is unauthorised appropriation of another person’s speech). This type of justification would also suggest against copyright protecting facts or data *per se.*

159 Drexl (2018), p. 87.

160 Mylly (2021). p. 1320 observes that overlaps between copyright and trade secrets protection have attracted much less attention than other IP overlaps.

161 *E.g.*, *see* Reichman and Samuelson (1997) (prior to the Directive’s adoption) and Davison and Hugenholtz (2005) (after the first CJEU rulings on the database right). More generally, *see* Synodinou (2019).

162 *See* Drexl (2018), pp. 68–85; Leistner (2021a), p. 387 et seq. *See also* European Commission (2018a) (“Study 2018”).

163 Study 2018, p. 27.

164 *Ibid*, pp. 25–44.

165 Leistner (2021a), pp. 396–398.

166 Drexl (2018), p. 82.

167 Such as *Directmedia Publishing GmBH v. Albert-Ludwigs-Universitat Freiburg* Case C-304/07. ECLI:EU:C:2008:552; and *Innoweb BV v. Wegener ICT Media BV* Case C-202/12, ECLI:EU:C:2013:850.

168 C-762/19, ECLI:EU:C:2021:434. For a discussion of the impact *see* Derclaye and Husovec (2021).

169 *CV-Online Latvia,* paras. 44 and 46.

170 Drexl et al. (2022), paras. 256–266; Opinion of the European Copyright Society on selected aspects of the proposed Data Act, 12 May 2022; Noto La Diega and Derclaye (2022).

171 Commission of the European Communities (2005), paras. 1.4 and 4.2 indicating that database production had fallen back to pre-directive levels and that the economic impact of the *sui generis* right had not significantly improved the competitiveness of the European database industry, and Study 2018, ii stating that the “effectiveness of the sui generis right, as a means to stimulate investment in databases, remains unproven and still highly contested” and at (iii), indicating that “most stakeholders have experienced low, if any, benefits from the Database Directive”.

172 Study 2018, (vii).

173 Derclaye and Husovec (2021).

174 Study 2018, vii.

175 *E.g.*, *see* Arts. 3 and 4 of the DSM Directive related to text and data mining, Art. 5 relating to cross-border teaching activities, and Art. 6 preservation of cultural heritage.

176 Leistner (2021a), pp. 396–398.
